# Poor sleep quality as a potential trigger for cognitive deficits

**DOI:** 10.1002/alz.087261

**Published:** 2025-01-03

**Authors:** Anna Carnes, Gerard Piñol‐Ripoll, M Ariza, N Cano, Barbara Segura, Carme Junqué, M Garoellda

**Affiliations:** ^1^ Cognitive Disorder Unit, Hospital Universitari Santa Maria, Lleida Spain; ^2^ Hospital Universitari Santa Maria de Lleida, IRBLleida, Lleida Spain; ^3^ Universitat Barcelona, Barcelona, Barcelona Spain; ^4^ Consorci Sanitari Terressa, Terrassa, Barcelona Spain; ^5^ Medical Psychology Unit, Institute of Neurosciences, University of Barcelona, Barcelona, Barcelona Spain

## Abstract

**Background:**

In the present study we aimed to assess the cognition of post‐COVID condition (PCC) participants regarding their sleep quality, and to analyse different possible moderators of this effect, such as quality of life (European Quality of Life‐5 Dimensions, EQ‐5D), fatigue (Chadler Fatigue Questionnaire, CFQ), cognitive reserve (Cognitive Reserve Questionnaire, CRC), and subjective cognitive complaints (Memory Failures of Everyday Questionnaire, MFE‐30).

**Method:**

We included 373 individuals with PCC and 126 healthy controls (HCs) from the NAUTILUS Project (NCT05307549 and NCT05307575) that were assessed with a comprehensive neuropsychological battery and different questionnaires.

**Result:**

We included 373 individuals with PCC and 126 healthy controls (HCs) from the NAUTILUS Project (NCT05307549 and NCT05307575) that were assessed with a comprehensive neuropsychological battery and different questionnaires. We found that PCC participants with poor sleep quality have a 4.3% greater risk of having immediate verbal memory deficits the greater the MFE‐30 score (Odd Ratio (OR) 1,043; Confidence Interval (CI) 1,023‐1,063). Also, they multiply their risk of having immediate verbal memory disorders by 2.4 when the EQ‐5D is low (OR 0,33; CI 0,145‐0,748), and they have less risk of having delayed visual memory deficits the greater the CRC (OR 0,963; CI 0,929‐0,999). With processing speed performance, PCC participants and poor sleep quality have a 6.7% greater risk of having deficits the greater the MFE (OR 1,059; CI 1,024‐1,096) and the risk of slowing processing speed triples the lower the EQ‐5D (OR 0,021; CI 0,003‐0,141).

**Conclusion:**

These results indicate that poor subjective sleep quality plays a role as a potential trigger for cognitive deficits. Therapeutic strategies to maximize sleep quality would be reducing not only sleep disturbances, but perhaps also cognitive impairment in PCC participants.

